# Physics-Based Protein Networks Might Recover Effectful Mutations—a Case Study on Cathepsin G

**DOI:** 10.1021/acs.jpcb.4c04140

**Published:** 2024-10-02

**Authors:** Fabian Schuhmann, Heloisa N. Bordallo, Weria Pezeshkian

**Affiliations:** † Niels Bohr International Academy, Niels Bohr Institute, University of Copenhagen, Blegdamsvej 17, 2100 Copenhagen, Denmark; ‡ Niels Bohr Institute, University of Copenhagen, Universitetsparken 5, 2100 Copenhagen, Denmark

## Abstract

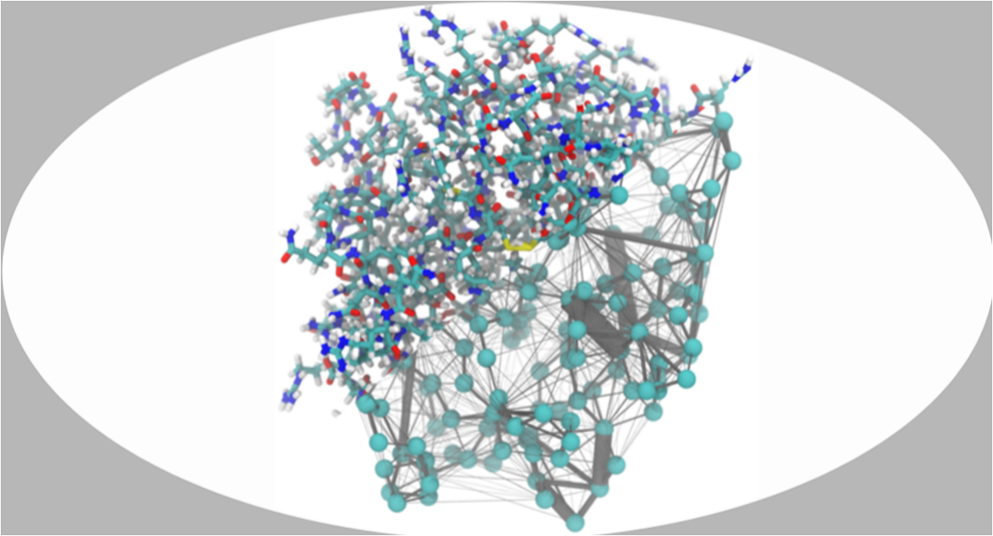

Molecular dynamics simulations have been remarkably effective for observing and analyzing structures and dynamics of proteins, with longer trajectories being computed every day. Still, often, relevant time scales are not observed. Adequately analyzing the generated trajectories can highlight the interesting areas within a protein such as mutation sites or allosteric hotspots, which might foreshadow dynamics untouched by the simulations. We employ a physics-based protein network and propose that such a network can adequately analyze the protein dynamics. The analysis is conducted on simulations of cathepsin G and neutrophil elastase, which are remarkably similar but with different specificities. However, a single mutation in cathepsin G recovers the specificity of neutrophil elastase. The physics-based network built on the interactions between residues instead of the distances can pinpoint the active triad in the proteins studied. Overall, the network seems to capture the structural behavior better than purely distance-based networks.

## Introduction

Protein structure’s conformation is directly linked to its function and state. A conformational change can mark the change from an active to an inactive state or *vice versa*, indicate the effects of bound ligands to the protein, or facilitate signaling pathways to downstream processes.^1−3^ Importantly, a conformational change does not necessarily happen at the site of the perturbation. For instance, Shahu et al.^4^ show that a single mutation in the bovine rod outer cone guanylate cyclase type 1 protein structure leads to a conformational change and rearrangements of two protein domains away from the mutation, which moves the protein to an always-active state associated with various diseases. Similarly, a slight change of charge in a cofactor bound inside the pigeon cryptochrome 4 protein results in distinct conformational changes at different sides of the protein, as discussed by Schuhmann et al.^2^ These long-range reactions to perturbation in the protein structure are often associated with the allostery of the protein or, in other words, the chemical information pathways along the amino acid residues in the protein structure, which then leads to the observed changes. However, even if the perturbed site and the reacting site are known, the path the information took through the protein might not be readily accessible. Furthermore, networks have been considered to combine and interpret experimental findings and computer simulations on the same level,^5^ or networks have been used to study proteins in a membrane environment.^6^

**Figure 1:**
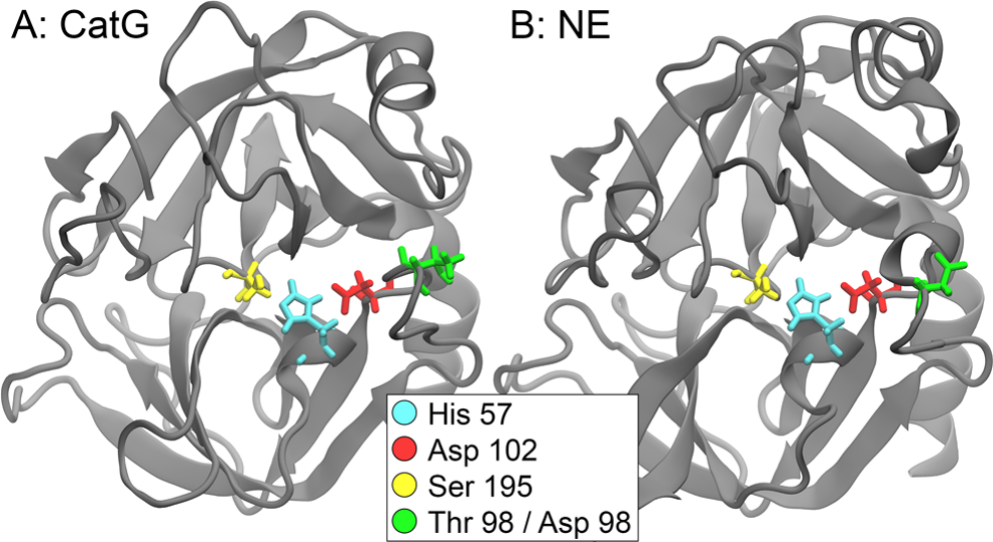
CatG (panel A) and the NE (panel B) protein structures are shown in their secondary structure representation. The highlighted residues are the active site (His 57, Asp 102, and Ser 195) in cyan, red, and yellow, respectively. The green residue is Thr 98/Asp 98, which was subjected to a mutation in CatG and has been proven to influence CatG’s specificity experimentally.^10^

The analysis of such networks becomes increasingly subtle, as on the other end of the spectrum, two proteins with only 32% sequence identity exhibit an almost identical crystal structure but different specificity. Two serine protease enzymes give such a case, neutrophil elastase (PDB ID 1ela, NE)^7^ and cathepsin G (PDB ID 1cgh, CatG).^8^ Both proteins have the same three amino acid residues in their center, which are denoted as the active triad needed for the serine protease but fulfill different tasks; NE can degrade the *Shigella* virulence factor, while CatG cannot. The structures are visualized in Figure 1. In an earlier study,^9^ conformational differences between the two protein structures were analyzed, showing a peculiar difference in the active triad of the proteins only accessible through molecular dynamics (MD) simulations. Furthermore, the mutation of a single amino acid residue (T98N) in cathepsin G enables the enzyme to cleave *Shigella*.^10^ Therefore, we are provided with two distinctly different yet similar proteins, and we know from experiments that they share an active triad and have pinpointed a critical mutation. Here, the primary focus is set on the nonmutated CatG structure as derived by Hof et al.^8^ to probe if one could have suggested the mutation based on the simulation alone. The not-mutated structure is termed the wild-type CatG.

If the proposed approach can identify pathways and suggest the known mutation and the active triad in NE and CatG, a validation is given, and more reasonable mutations might be suggested. Suggesting the right mutations or mutation sites computationally might allow more thorough and economically feasible experimental studies of these mutations and thus allows the generation of more targeted results.

In order to detect these pathways, networks are constructed from the protein structure, which often associates a single amino acid residue with a node (vertex) in the network, while two amino acid nodes are considered to have a connecting edge if some condition is met. Often, the distance between the backbone atoms or the center of mass/geometry of the amino acids is considered and endowed with a threshold. For instance, Kattnig et al.^11^ call two amino acids connected if their centers of mass are less than 8 Å apart. Another approach, employed by Wang et al.^12^ in their program Ohm, considers the distance between every atom of a residue to every atom of the other residue and counts the number of atoms that are within 3.5 Å of each other to determine the edge and associated edge cost. Most of the approaches are purely distance-based and often lack information regarding the physical strength or other attributes of a connection. Even network approaches dealing directly with nonbonded interactions often rely on distances and geometries.^13^ Here, to remedy the limitations described above, we have developed a network based on the potential energy employed within the MD framework. Therefore, the network includes not only distances but also the strength of the interaction, e.g., van der Waal forces and electrostatics. This tracks both the underlying topology of the protein structure and captures the realistic physical interaction of the atoms. This approach also allows catching perturbations over time if the simulation was, for instance, subjected to steered MD. Furthermore, no threshold value is needed, making the resulting network less dependent on the individual choice of cutoff. Once the network graph is created, a variety of methods can be employed to investigate the allosteric pathways. For instance, the shortest paths between vertices can be analyzed and particularly centralized amino acids might be found by counting the number of shortest paths going through that amino acid’s network vertex.^11^ A second method entails considering the network as an adjacency matrix, which contains information about all edges and their costs in a network; one can directly calculate the number of paths of length *n* from one vertex to another by considering the matrix exponential, as has been rigorously analyzed by Estrada et al.^14^ and used on a distance-based network of SARS CoV-2 main protease. A general review on graphs and pathfinding within graphs is given by Das et al.^15^ Here, we employ shortest path algorithms to probe the potential energy-informed protein network to extract information about active sites or estimate the location of effects induced by a perturbation. With this first study on CatG and NE utilizing the potential energy network approach, we aim to open the door for more validation and tests to probe the network type as a potential predictive tool for mutation sites.

## Methods

### Molecular Dynamics

The simulation trajectories for the NE and the nonmutated CatG (wild type) MD simulations are taken from an earlier study.^9^ For NE and CatG, the residue numbering follows the chymotrypsinogen numbering introduced by Blow et al.^16^ The first snapshot of the CatG simulation serves as a starting structure for a mutated CatG simulation, in which T98N the threonine at residue position 98 is replaced with an asparagine. The mutated simulation is denoted by CatG T98N. The mutation is done using the mutator plug-in in VMD^17^ and subsequently equilibrated and simulated. The simulation is conducted through the online platform VIKING,^18^ which employs the simulation software NAMD^19,20^ with the CHARMM36 forcefield with CMAP corrections.^21−28^

The system is equilibrated in a three-step process, followed by the production simulation. The first equilibration stage includes an energy minimization step and 1 ns of MD simulation. The simulation is conducted in an NPT ensemble (constant number of particles, pressure, and temperature), while only water and ions are free to move. Lifting some restraints, in equlibration stage 2, 2 ns are simulated. The side chains of the protein structure are free to move now. In equilibration stage 3, another 2 ns is simulated with all restraints lifted. Furthermore, the simulation switched to an NVT ensemble (constant number of particles, volume, and temperature). For all three equilibration stages, the integration time step is set to 1 fs and the temperature is 315 K.

The production simulation is conducted for 400 ns in an NVT ensemble with an integration time step of 2 fs. In the production simulation, the bond lengths containing hydrogens are restrained. The temperature is set to 315 K. Electrostatic interactions were treated with particle-mesh Ewald (PME) with a short-range cutoff 12 Å, and van der Waals interactions were switched off smoothly between 10 and 12 Å. An overview of the three simulations can be seen in Table 1 and an assessment of their stability is shown by the root-mean-square deviation in the Supporting Information, S2.

**Table 1:** Three Types of MD Simulations Are Listed and Named with Their Corresponding Simulation Length

structure	mutation	simulation length (ns)	denoted as
CatG		400	CatG/CatG wild type
CatG	T98N	400	mutated CatG/CatG T98N
NE		400	NE

The interaction energies are calculated with the same program and parameters used for the MD simulation, here NAMD.^20^

### Network Generation

We chose three different network generation concepts, which rely on distances and have been previously used for MD trajectory analysis. The results of the network approaches are then compared to those of the proposed energy-based network. The distance-based approaches either rely on the whole residue, employed earlier by Kattnig et al.,^11^ or single atoms within the residue, as proposed by Wang et al.^12^ The third network is derived from the potential energy obtained from the MD simulations. Details are explained in the following paragraphs.

#### Residue Based

For each simulation snapshot in the MD trajectory, the center of mass is calculated for each individual residue. For each pair of residues, the distance between their centers of masses is considered and averaged over all simulation snapshots. We generate a symmetric *N* × *N* matrix, with *N* being the number of amino acid residues. From that matrix, two variations of the adjacency matrix are generated.

The first variation yields a graph with uniform edge costs. For each entry, if the averaged distance between the centers of mass is below or equal to 8 Å, the value is set to 1, and 0 otherwise. In the second variation, if the distance is >8 Å, the value is set to 0. The distances below 8 Å remain unchanged, and the distance is associated with the respective edge as a cost.

#### Atom Based

The network is generated from the final snapshot of the MD simulation trajectory. For each pair of residues, the atomic distances from all atoms *a*_*i*_ in one residue *A* are calculated to all atoms *b*_*j*_ in residue *B* with *i* ∈ {1, ···, *I*}, *j* ∈ {1, ···, *J*}, and *I*, *J* being the number of atoms in residue *A* and *B*, respectively. If the distance between two such atoms is less than 3.5 Å, then the connection is counted. The number of such connections is denoted as *c*. The connectivity from residue *A* to *B* is then , which is then associated to the edge from residue *A* to residue *B*. Analogously, the connectivity from residue *B* to *A* is . This results in a nonsymmetric matrix and thus in a directed network.

For shortest path analyses, the edge costs are inverted, such that a higher connectivity would lead to a lesser edge cost.

#### Potential Energy Based

The network based on the potential energy requires some additional calculations based on the MD simulations. For each pair of residues in the MD simulation, NAMD energy^19^ is run to calculate the potential energy contribution the amino acids experience between each other. This procedure requires  executions of NAMD energy, with *N* being the number of amino acids in the sequence of the protein structure. The number of necessary runs can be further reduced by only considering amino acid pairs, whose shortest distance between atoms is greater than the cutoff value for nonbonded interactions, which is chosen for the MD simulation. Overall, the parameters for NAMD energy align with the parameters used for the production simulations.

The program yields the unbonded potential energy between two residues for every simulation snapshot. This potential energy includes van der Waals and electrostatic interactions. The nonbonded interactions depend on the distance between the two residues. Therefore, the distance between residues is still part of the network but is augmented by the physical properties of the MD simulation.

**Figure 2:**
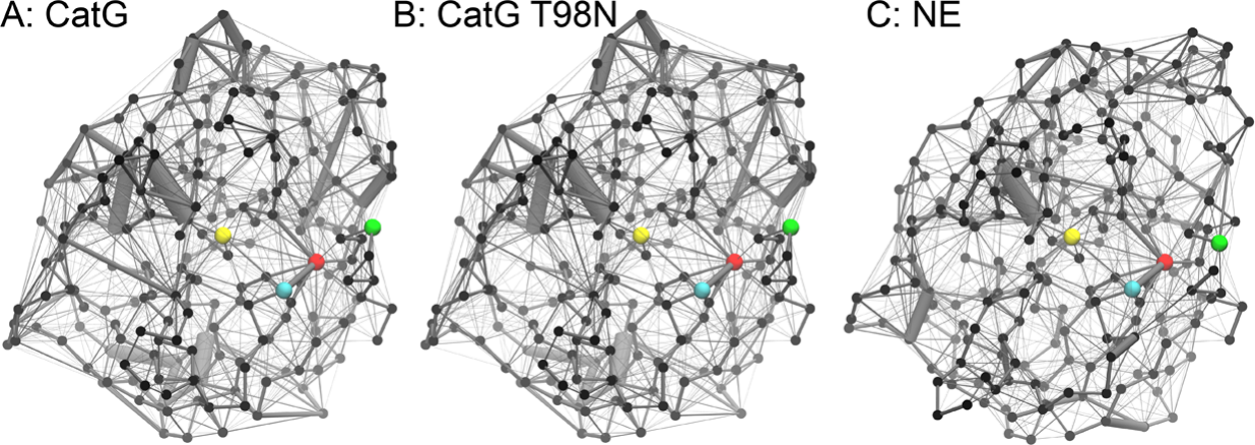
The panels show interaction potential energy network mapped onto the protein structures of CatG, CatG T98N, and NE. The width of a connection is proportional to the interaction potential energy between the connected amino acid residues. For orientation, the active triad and the mutation sites are colored analogously to Figure 1, with His 57 in cyan, Asp 102 in red, Ser 195 in yellow, and residue 98 in green.

The nonbonded potential energy term includes:^19^
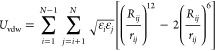
1
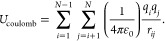
2*r*_*ij*_ is the distance between two nonbonded atoms *i* and *j* and  is the equilibrium distance between the two atoms. ρ_*i*_ is the van der Waals radius for atom *i* and  is the potential minimum influenced by the interacting atoms *i*, *j*. *q*_*i*_, *q*_*j*_ are the charges of the respective atoms, and ε_0_ is the vacuum permittivity. The terms are calculated for all pairs of atoms in the regular simulation. In NAMD energy, the potential energy is explicitly calculated for interactions from one residue to another. The calculations are aligned with the simulation parameters and the forcefield. Hence, nonbonded interactions are not calculated between bonded atoms, and some adjustments to the potential are introduced based on the CHARMM36 forcefield with CMAP corrections^21−28^ to angular and dihedral atoms. The resulting network was mapped onto the protein structures in Figure 2 for visualization purposes. The greater the radius of the drawn connection, the greater the absolute value of the interaction potential energy between the two residues.

In order to translate the calculated interaction potential energy to the network, each edge between two residue vertices is then associated with the inverse of the mean of the absolute values of the potential energies for each simulation snapshot. For the network, it is disregarded whether the energy would lead to a repulsive or attractive contribution, and only its strength is considered. The inverse of the mean is chosen, so the shortest path algorithms can be applied.

### Network Analyses

The different generated networks are all subjected to a shortest path analysis, in which the average shortest path length from one residue to all of the others is considered. The shortest path is calculated using Dijkstra’s algorithm,^29^ which results in a square matrix containing the pairwise shortest paths’ length between all residues. The average over the matrix’s column associated with a particular residue is then the average shortest path length. The lower the value for a given residue, the better connected it is, and other residues can be reached more swiftly. A perturbation in amino acid residues with a low average shortest path is thus considered more effectful or disruptive on the protein structure.

## Results and Discussion

The different network generation approaches were employed to study the behavior of NE and CatG based on their MD simulations. CatG was simulated in the wild type and a mutated form. Figure 1 shows the secondary structure of CatG and NE with the active residues (cyan, red, yellow) and the mutation site (green) suggested by Averhoff et al.^10^ highlighted.

In the residue distance-based network, the shortest path most notably sorts the amino acid residues from the center of the protein to the surface residues. The behavior is encouraged by the overall ball-like form of the studied protein structures. The active triad is located in the center of the protein and is thus among the residues with a shorter average path length to the other residues. The active triad is, however, indistinguishable from other buried amino acid residues. The known mutation site shows a longer average path length in the CatG simulations than in the NE simulation.

The average shortest path length also sorts the amino acid residues from the center to surface in the atom distance-based network. However, the best-connected residues are more pronounced than the residue distance-based network. The active triad is more visible. In all simulations, two out of three amino acid residues of the active triad show the overall minimal average shortest path length. The discovered mutation site at residue 98 shows bad connectivity in the CatG simulation but is not clearly distinguishable from other poorly connected residues on the surface. Interestingly, in CatG T98N and NE simulations, the mutated residue is connected better and vanishes in the average of the protein structure.

Finally, in the potential energy-based network, the amino acid residues no longer show a clear trend of being sorted from the center to the surface, even though the distances directly influence the interaction energies. Furthermore, the active triads are the best-connected amino acid residues in both CatG simulations and are within the best four connected residues in the NE simulation. Overall, the paths are longer in the NE simulation, which could indicate that the interaction energies are generally weaker, which can be observed in the raw numbers. CatG has an average absolute interaction energy of 0.82 kcal/mol, a CatG T98N of 0.84 kcal/mol, and NE shows an average of 0.68 kcal/mol.

**Figure 3:**
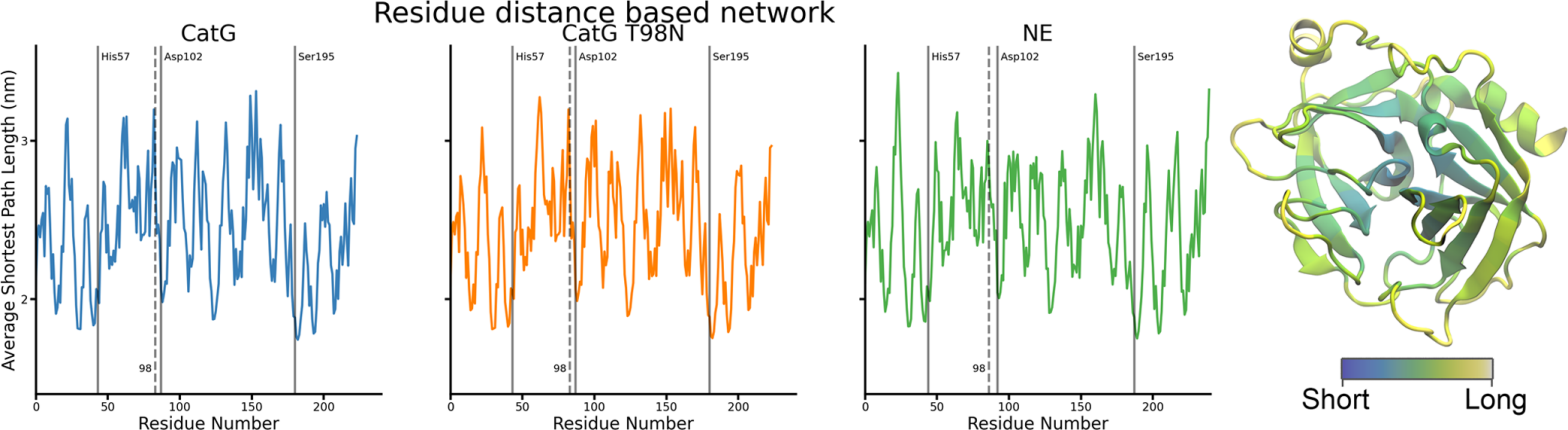
Panels show the average shortest path per residue length relative to all other residues for the different MD simulations conducted for the residue distance-based network. For all conducted simulations, the shortest path length through the residue distance-based network sorts the amino acid residues from central to surface positions in the protein structure. A visualization of the sorting behavior is shown on the structure of CatG, in which short paths are bundled in the center (blue), while longer paths spin outward toward the surface (yellow).

**Figure 4:**
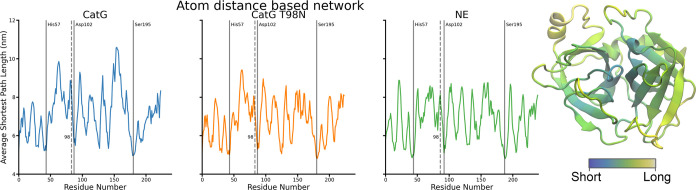
Panels show the average shortest path per residue length relative to all other residues for the different MD simulations conducted for the atom distance-based network. Like the residue distance-based network, the amino acids are sorted according to their centrality in the protein structure. However, the active triad is shown in a more pronounced manner. A visualization of the sorting behavior is shown on the structure of CatG, in which short paths are bundled in the center (blue), and longer path residues (yellow) are located more toward the surface. However, the spread is less clear than that of the residue distance-based network.

**Figure 5:**
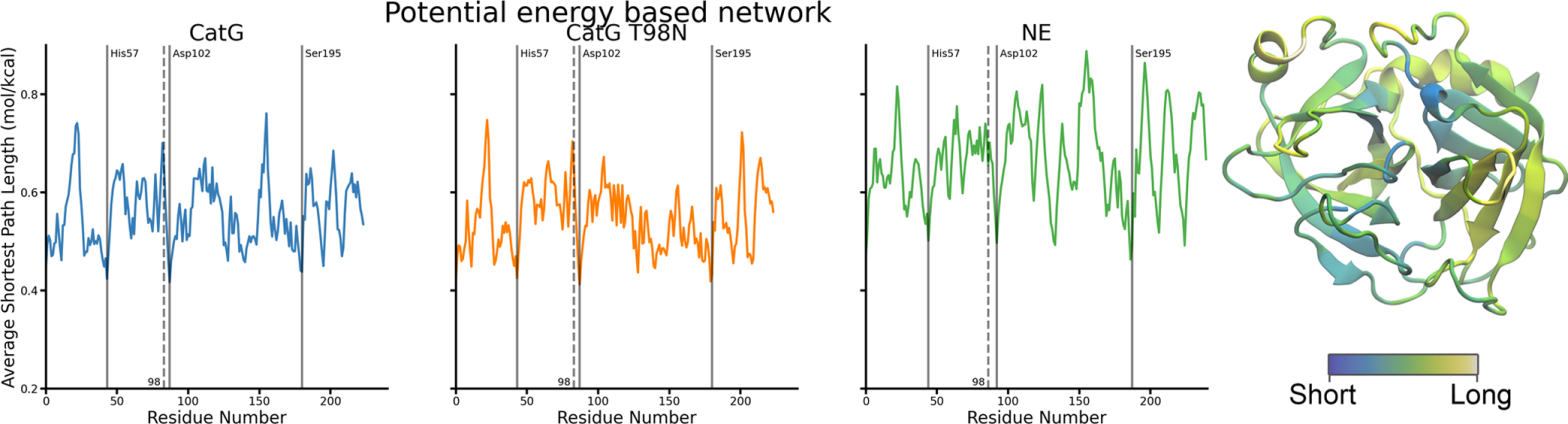
Panels show the average shortest path per residue length to all other residues for the different conducted MD simulations for the network based on the interaction potential energy. For both simulations, wild type and mutant, of CatG, the active triad can be identified as the three residues with the minimal path length. NE does not show such a clear distinction. The mutation site is among the top three longest paths in the CatG simulations but is not noticeable in the NE network. The shortest paths through the energy-based network do not show an apparent sorting behavior based on the residue’s location within the protein structure as visualized in the structure of CatG.

**Figure 6:**
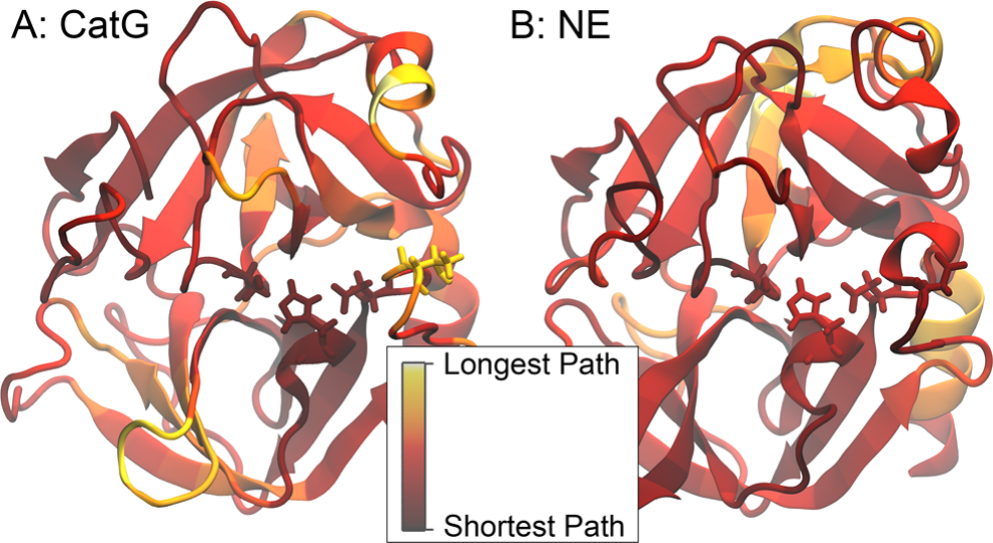
Differences seen in Figure 5 are mapped onto the protein structure, with the active triad and residue 98 highlighted. In a direct visual comparison, it can be seen that residue 98 is particularly poorly connected in CatG, while its surrounding residues are relatively better connected. In the NE structure, residue 98 is relatively well connected and exhibits a short average shortest path length. The active triad exhibits a short average shortest path length in both structures.

The mutation site is within the three worst connected residues in the CatG simulations and is not noticeable in the NE simulation. The other two peaks correspond to Ala 37 and Phe 172. Both residues are in areas where NE has been mutated to be CatG-like.^10^ It turned out that the mutations around Ala 37 allowed NE to fulfill both the NE function and the CatG function, while the mutations around Phe 172 showed a limited specificity like the CatG wild type. While there are no mutation studies in CatG to imitate NE in these regions, they might be viable test sites based on the network analysis and the previous reversed experiments. In order to visually compare the CatG simulation with the NE simulation, the average shortest path lengths have been mapped onto the respective structures analogously to the structure representations shown in Figures 3–5. The resulting structures are shown in Figure 6. The visually most significant difference seems to show in the mutation site at residue 98, which is colored yellow in the CatG structure but red in the NE structure. Other regions follow a similar trend.

**Figure 7:**
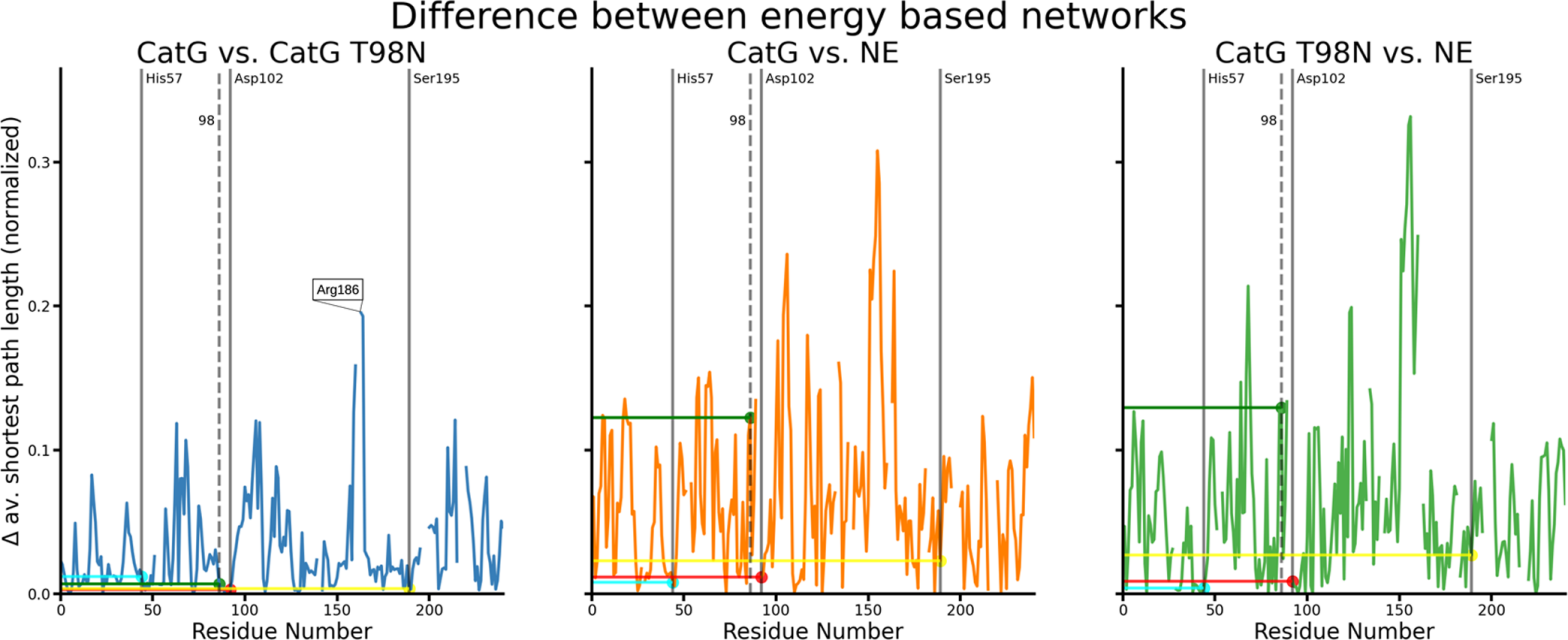
Differences seen in Figure 5 are quantified for each pair of structures. The plots show the absolute difference between the normalized average length and shortest path per residue for the compared structures, respectively. As extensively discussed earlier^9^; the CatG and NE structures exhibit a different number of amino acid residues. In order to meaningfully compare residues, the residues are compared according to a sequence alignment.^30^ The gaps indicate residues without a partner in the alignment. Overall, the shortest paths’ lengths are more similar between the CatG wild type and mutant than toward the NE structure. At the same time, there are noticeable differences in the active triad when comparing the CatG to NE structures. The difference in path length for the active triad and residue number 98 is additionally highlighted by the colored horizontal lines. His 57 is cyan, Asp 102 is red, Ser 195 is yellow, and residue 98 is marked green.

Quantitatively comparing the CatG and NE results comes with a cavity. As the sequences of the protein structures do not share the same length, a direct one-to-one comparison of the residues can not be attempted. In order to circumvent the problem, the sequences of CatG and NE were aligned,^30^ and only the aligned pairs were compared. Amino acid residues without a partner are ignored. The procedure introduces gaps in the sequence and thus artificially stretches the number of residue indexes to 241, while by themselves, CatG and NE have 224 and 240 amino acid residues, respectively. The alignment is shown in the Supporting Information, S5. A more thorough discussion of the implications of different lengths of amino acid sequences between CatG and NE has been undertaken in an earlier study.^9^ The resulting comparison can be seen in the differences in Figure 7. Additionally, a comparison between CatG and CatG T98N without the adjustment to the sequence length can be found in the Supporting Information (Figure S3).

Compared to the differences between the CatG and NE simulations, the similarity between the two CatG networks, wild type and mutant, is striking, particularly in the focused active triad and the mutation site. The most remarkable difference for the CatG networks is exhibited by residue 186 (using the counting scheme by Blow et al.^16^). The residue is right at the tip of a loop, extending outward from Ser 195, and is surface exposed. The area of CatG has been reported as necessary for the specificity of the protein in identifying targets.^8,31^

The differences with respect to NE are more severe. Unsurprisingly, the mutation site shows a great difference, as can already be seen in Figure 6, with Asp 98 being poorly connected in CatG and better connected in NE. The slight change in position may also give rise to the differences. Concerning the active triad, the differences become smaller for His 57 and Asp 102 once the mutation is introduced, while the difference in Ser 195 increases. Following an earlier conformational study,^9^ it was suggested that a slight difference in Ser 195 might influence the specificity of CatG and NE as it forms hydrogen bonds with the target to be cleaved by the serine protease. Overall, the average normalized difference over all residues decreases from 0.069 in the CatG vs NE difference to 0.062 in the CatG T98N vs NE difference. The magnitude of the differences is more than 2-fold the standard deviation for the CatG WT network obtained from individual snapshot energies from the MD simulation. Further visualization is shown in the Supporting Information, Figure S4.

Finally, a comparison between the different network types is in order. Here, we focus on the example of the mutation site and claim that the potential energy network points toward the mutation site as a significant peak in the CatG simulations, while the distance-based networks do not. This can be interpreted by considering the ratio of the peak at residue 98 to the average shortest path length across all residues. The value has been normalized to account for the differences in the unit and the results are shown in Table 2, where the distance-based networks show a smaller peak in the mutation site of residue 98 compared to the mean than its counterpart in the energy-based network. Interestingly, the mutation site relatively vanishes in the atom distance-based network for CatG T98N. While the mutation site is greatly reduced in its peak in the energy-based network for NE, the peak ratios are the highest in both CatG simulations compared to the other network generation approaches.

**Table 2:** Ratio between the Value for Residue 98 and the Mean of the Shortest Path Lengths for All Network Generation Approaches, Normalized to be Able to Compare between the Different Units

ratio peak/avg.	CatG	CatG T98N	NE
residue distance	1.50	1.52	1.55
atom distance	1.51	1.18	1.63
energy based	1.70	1.74	0.95

## Conclusion and Outlook

Considering the two proteins neutrophil elastase (NE) and cathepsin G (CatG), the combination of molecular dynamics simulations and graph network approaches showed crucial residues within the protein structures. For instance, based only on the simulation of the wild type of CatG, the potential energy network showed the active triad in the protein as the best-connected amino acid residues. On the other end of the connectivity spectrum, residue 98 is among the three least connected residues. Residue 98 is a known mutation site in CatG, which bestows the specificity of NE onto CatG. Our approach suggests a mutation or perturbation of Ala 37 and Phe 172 in the CatG structure that might lead to a major functional change in CatG, potentially also recovering the NE specificity, as they are both poorly connected to the other residues but show a similar value to the known mutation in residue 98. Additionally, both sides are in locations where mutations in NE were performed to make the behavior CatG-like. Such mutations could be performed experimentally to validate the network approach.

Pinpointing the mutations in NE and CatG and probing the effects on the specificity of the proteins experimentally has been done earlier,^10^ in which numerous costly mutations were introduced in the wet lab and the results were measured. If a functioning prototype of a computational mutation prediction algorithm had been known, then the costs for the experiments might have been significantly reduced. Knowing the interesting mutations, however, allows the validation of such an algorithm on their example.

In the specific example of NE and CatG, we showed the differences in results obtained from a potential energy-generated network compared to more traditional distance-based methods, which already led to a more clear-cut identification of essential residues and might hint toward a predictive capability for mutation sites if tested and validated more rigorously. While the method presented in this study offers a promising path to qualitative protein network analysis and unraveling allosteric connections, it is important to notice several limitations. First, the computational costs of generating the potential energy-based networks are still significant, and it is not straightforward to test arbitrary protein molecular dynamics simulations. This limitation leads to difficulties in validating the approach on other proteins to empirically test the merit of the potential energy network for protein structures. These drawbacks might be addressed through preliminary statistics and a reduction of considered simulation snapshots for the network or by the generation of an automated workflow that can be applied to different protein structure simulations.

While overcoming the limitation, one might use the approach in future works on simulations with proteins and ligands or to detect binding sites with promising attributes, as there are no requirements for the number of atoms in a residue. Furthermore, a network might be employed to rank potential bound ligands based on their effect on the protein receptor and, thus, its function. Additionally, if the workflow can be made more efficient, generating potential energy networks of whole protein complexes or protein-membrane combinations becomes conceivable.

In summary, the potential energy network, which still contained atomistic distances implicitly, seems to capture changes in structure and behavior better than a distance-based network. While the network analysis contained an abundance of noise in all differently generated networks, the potential energy network still captures more subtle and more dynamic differences. We, therefore, propose a continued study of networks generated from different physical, chemical, or biological principles and the combination of these attributes. It is even questionable if the connection from residue A to residue B should have the same cost as the connection from residue B to residue A. In this study, only the atom distance-based network formed such a directed network, even though it captured similar notions as the straightforward residue distance network approach. Such a non-Euclidiean network might allow a rigorous analysis of allosteric processes without the need for artificially chosen thresholds and cutoffs, which had to be employed in the distance-based network generations.

## Supporting Information Available

Example of a shortest path through a network (Section S1); root mean square deviation for MD simulations (Figure S2); difference between CatG and CatG T98N average shortest path length for the potential energy network (Figure S3); standard deviation across the trajectory (step 1000) of the CatG WT shortest path length for the energy network (Figure S4); sequence alignment between CatG and NE (Table S5) (PDF)
