# Supplementary material for: Physics-Based Protein Networks Might Recover Effectful Mutations—a Case Study on Cathepsin G
Source: J Phys Chem B. 2024 Oct 2;128(41):10043–50. doi: 10.1021/acs.jpcb.4c04140 (PMC11492240; doi:10.1021/acs.jpcb.4c04140)
Supplement: Supplementary file 1 — jp4c04140_si_001.pdf [file jp4c04140_si_001.pdf]

Physics Based Protein Networks Might Recover Effectful Mutations – a Case Study on Cathepsin G

Fabian Schuhmann,[*,†] Heloisa N. Bordallo,[‡] and Weria Pezeshkian[†]

†*Niels Bohr International Academy*

Niels Bohr Institute

University of Copenhagen

Blegdamsvej 17

2100 Copenhagen, Denmark

‡*Niels Bohr Institute*

University of Copenhagen

Universitetsparken 5

2100 Copenhagen, Denmark

E-mail: fabian.schuhmann@nbi.ku.dk

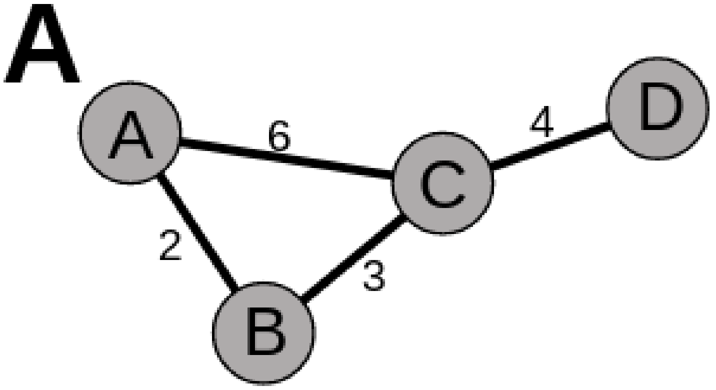

B

	A	B	C	D
**A**	0	2	6	0
**B**	2	0	3	0
**C**	6	3	0	4
**D**	0	0	4	0

Figure S1: Consider a protein structure containing four residues (A, B, C, D) and a corresponding made up network. **A:** An example network is shown with four residues denoted A, B, C, and D with corresponding edge costs. **B:** The same network is shown in its matrix form.

Supporting Information Available

S1: Shortest path example

Figure S1 shows, pictographically, a simplied example network with residues and their connections. Each connection has an edge cost describing how favorable that connection is. The network (or its matrix representation) can then be used to calculate, for instance, the average length shortest path for each residue.

In the example, the shortest path from A to B is 2, denoted as $p(A, B)$. The length of the

average shortest path for all residues in the example is then

$$\begin{aligned}
s(A) &= \frac{1}{N} \sum_{X \in \{A,B,C,D\}} p(A, X) = \frac{1}{N}(0+2+5+9) = 4 \\
s(B) &= \frac{1}{N}(2+0+3+7) = 3 \\
s(C) &= \frac{1}{N}(5+3+0+4) = 3 \\
s(D) &= \frac{1}{N}(9+7+4+0) = 5,
\end{aligned} \tag{1}$$

with N being the number of residues (here, $N = 4$). The resulting value is then plotted for each residue. Note, that the shortest path from A to C is actually the path passing B, even though a direct edge from A to C exists. Its cost is just too expensive.

Supplementary figures and tables

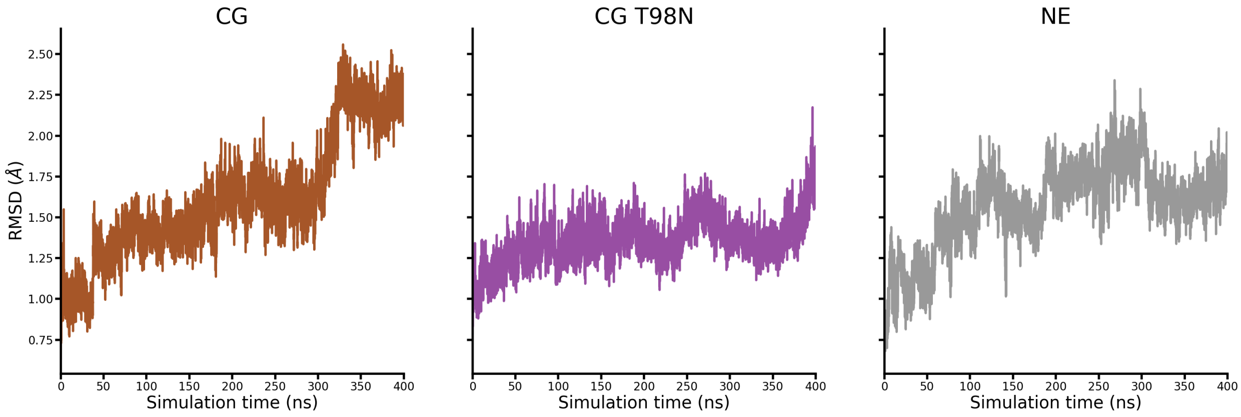

Figure S2: The root mean square deviation (RMSD) is shown for the three conducted MD simulations labeled as CatG for the wildtype CatG simulation, CatG T98N for the mutant simulation and NE for the neutrophil elastase simulation. The RMSD was calculated with a step size of 10 across 400 ns of simulation after each simulation was aligned to its first snapshot, respectively. The RMSD is below 2.5 Å in all simulations, which is within the range of the resolution of the crystal structures (1.8 Å for cathepsin G, 2.0 Å for neutrophil elastase).

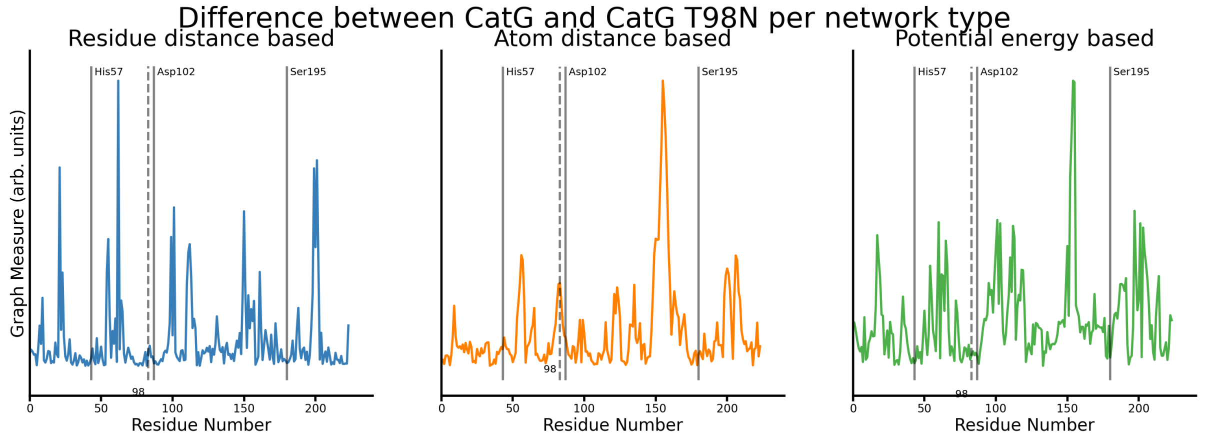

Figure S3: The difference per network type of the average length shortest path per residue between CatG and the mutant CatG T98N is shown. The atom based network shows a reorganization, difference in the mutation site directly, which is less pronounced in the residue distance based network. In the residue distance and atom based network, the differences in the active triad are hardly observable. In the potential energy based network, a distinctive difference is seen in the Ser 195, which corresponds to the findings of the earlier study, that the Ser 195 is the key to the difference in specficty.[9]

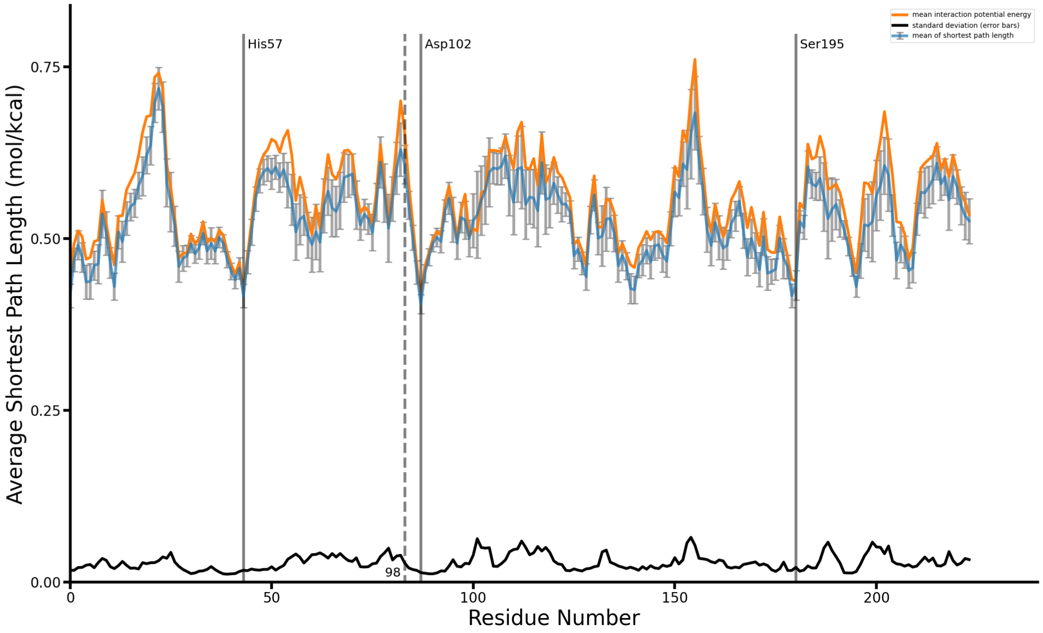

Figure S4: The network built from the mean of the interaction potential energy (as in the main manuscript) is shown, as well as the mean of the average shortest path lengths from equally spaced single snapshots from the molecular simulation trajectory. The standard deviation is shown as error bars, as well as a line plot. The mean standard deviation over all residues is 0.02.

Table S5: The sequence alignment between CatG and NE. Even though neither protein has 240 amino acid residues, the gaps in the alignment allow a feasible indexing until 240.

```
  NE    0   VVGGTEAQRNSWPSQISLQYRSGSSWAHTCGGTLIRQNWVMTAAHCVDRELTFRVVVGEH
CatG    0   IIGGRESRPHSRPYMAYLQIQSPAGQSR-CGGFLVREDFVLTAAHCWG--SNINVTLGAH

  NE   60   NLNQNNGTEQYVGVQKIVVHPYWNTDDVAAGYDIALLRLAQSVTLNSYVQLGVLPRAGTI
CatG   60   NIQRRENTQQHITARRAIRHPQYNQRTIQN--DIMLLQLSRRVRRNRNVNPVALPRAQEG

  NE  120   LANNSPCYITGWGLTRTNGQLAQTLQQAYLPTVDYAICSSSSYWGSTVKNSMVCAGGDGV
CatG  120   LRPGTLCTVAGWGRVSMR-RGTDTLREVQLRVQRDRQCLR--IFGSYDPRRQICVGDRRE

  NE  180   R-SGCQGDSGGPLHCLVNGQYAVHGVTSFVSRLGCNVTRKPTVFTRVSAYISWINNVIAS
CatG  180   RKAAFKGDSGGPLLCNN--------VAHGIVSYGKSSGVPPEVFTRVSSFLPWIRTTMRS

  NE  240   N
CatG  240   -
```
